# Immune-Molecular Link between Thyroid and Skin Autoimmune Diseases: A Narrative Review

**DOI:** 10.3390/jcm13185594

**Published:** 2024-09-20

**Authors:** Palma Carlucci, Federico Spataro, Mattia Cristallo, Mario Di Gioacchino, Eustachio Nettis, Sebastiano Gangemi

**Affiliations:** 1 Department of Emergency and Organ Transplantation, School of Allergology and Clinical Immunology, University of Bari Aldo Moro, 70126 Bari, Italy; palma.carlucci@uniba.it (P.C.); federico.spataro@uniba.it (F.S.); mattia.cristallo@uniba.it (M.C.); e.nettis@libero.it (E.N.); 2 Center for Advanced Studies and Technology (CAST), G. D’Annunzio University of Chieti-Pescara, 66100 Chieti, Italy; 3 Institute of Clinical Immunotherapy and Advanced Biological Treatments, 65100 Pescara, Italy; 4 Department of Clinical and Experimental Medicine, University of Messina, 98124 Messina, Italy; sebastiano.gangemi@unime.it

**Keywords:** autoimmune thyroiditis, skin autoimmune diseases, interleukin-23, oxidative stress, CTLA-4, CXCL9/10, JAK/STAT pathway

## Abstract

Autoimmune skin disorders, including Psoriasis, Lichen Planus, Vitiligo, Atopic Dermatitis, and Alopecia Areata, arise from a combination of genetic predisposition, external factors, and immunological dysfunction. It is well-documented that there is a strong correlation between autoimmune thyroid diseases and a range of dermatological disorders, especially urticaria. This review investigates possible links between autoimmune thyroiditis and a broader spectrum of autoimmune skin conditions, analyzing shared genetic markers, immunological mechanisms, and clinical correlations. Common pathogenic mechanisms include disrupted immune tolerance and oxidative stress, leading to chronic inflammation. Genetic factors, such as IL-23 receptor gene variants, increase the risk for Psoriasis, Alopecia Areata, and Hashimoto’s thyroiditis. Additionally, CTLA-4 mutations enhance susceptibility to autoimmune thyroid and skin disorders. Shared genetic susceptibility was also reported in Lichen Planus and Vitilgo, even if different genetic loci might be involved. The breakdown of the immune system can determine a pro-inflammatory state, facilitating the development of autoimmunity and auto-antibody cross-reactions. The presence of similar antigens in skin cells and thyrocytes might explain why both tissues are affected. The significant overlap between these conditions emphasizes the necessity for a comprehensive diagnosis workup and treatment. Future research should focus on clarifying specific immunological pathways and identifying novel biomarkers.

## 1. Introduction

The correlation between chronic idiopathic urticaria (CIU) and autoimmune thyroid diseases was proposed for the first time several decades ago. Bagnasco et al. [1] highlighted the possible explanations and associations between these two pathologies and the key role played by different autoantigen–autoantibody systems (anti-FceRI, anti-TPO) that synergize in generating C5a and triggering mast cells and basophils. On the one hand, TSH might drive the production of pro-inflammatory cytokines by lymphocytes and monocytes. On the other hand, viral and bacterial superantigens (*Staphylococcus aureus, Helicobacter pylori*, Hepatitis C virus) can induce relevant changes in the lymphocyte repertoire’s composition. As was conducted for CIU, we analyzed the genetic predisposition, the environmental triggers, and the cytokine pathways shared in the autoimmune responses between these five common skin diseases (Psoriasis, Lichen Planus, Atopic Dermatitis, Alopecia Areata, and Vitiligo) and thyroiditis [2]. Under normal circumstances, the human immune system acts as a vigilant defender, protecting the body from foreign invaders. However, in autoimmune diseases, this finely tuned system malfunctions, mistakenly attacking healthy tissues.

The thyroid gland is particularly susceptible to such attacks, with Hashimoto’s thyroiditis (hypothyroidism) and Graves’ disease (hyperthyroidism) being the most common autoimmune diseases. Interestingly, research suggests a potential association between these thyroid autoimmune disorders and the development of specific immunological cutaneous diseases. Their shared embryological origin and the expression of similar immunological markers could create a vulnerability. If a breakdown in immune tolerance occurs, the immune system, primed to recognize certain antigens (molecules on the cell surface) in the context of the shared origin, could launch an attack on both the skin and the thyroid. This concept provides a potential explanation for the observed association between thyroid autoimmunity and certain immunological cutaneous diseases. During the gastrulation stage of embryonic development, a single continuous sheet of cells, known as the ectoderm, forms the outermost layer of the developing embryo. From this layer, diverse tissues and organs are generated. As development progresses, the ectoderm undergoes a series of transformations, with specific regions giving rise to distinct structures.

One key event in ectoderm development is neurulation, which involves the formation of the neural tube (the precursor to the central nervous system) and the neural crest. The neural crest emerges as a population of migratory cells that detach from the developing neural tube. These highly versatile cells migrate throughout the embryo, contributing to the formation of various structures.

Notably, in the context of this discussion, the neural crest plays a pivotal role in the development of the thyroid gland while the neural crest contributes to the thyroid constitution, the remaining ectoderm undergoes its differentiation process. This ectoderm differentiates to form the outermost layer of the skin, the epidermis. The epidermis serves as the body’s primary barrier against the external environment, protecting us from pathogens, desiccation, and harmful UV radiation. It comprises a stratified epithelium with distinct cell layers, each playing a specific role in maintaining skin integrity [3].

Given the interconnected embryological development of the thyroid and skin, and their shared vulnerability to autoimmune attacks, this review aims to be a first step in understanding these links, in order to improve clinical decision making and potentially discover more targeted and effective treatments.

To investigate this intricate relationship, a comprehensive literature search was conducted using PubMed, Scopus, and Google Scholar databases. Additionally, relevant studies identified through reference lists were incorporated. The focus of the search was on original research articles, meta-analyses, and systematic reviews published in peer-reviewed journals.

The subsequent sections will delve into this relationship, highlighting the mechanisms and clinical implications of these associations.

**Figure 1:**
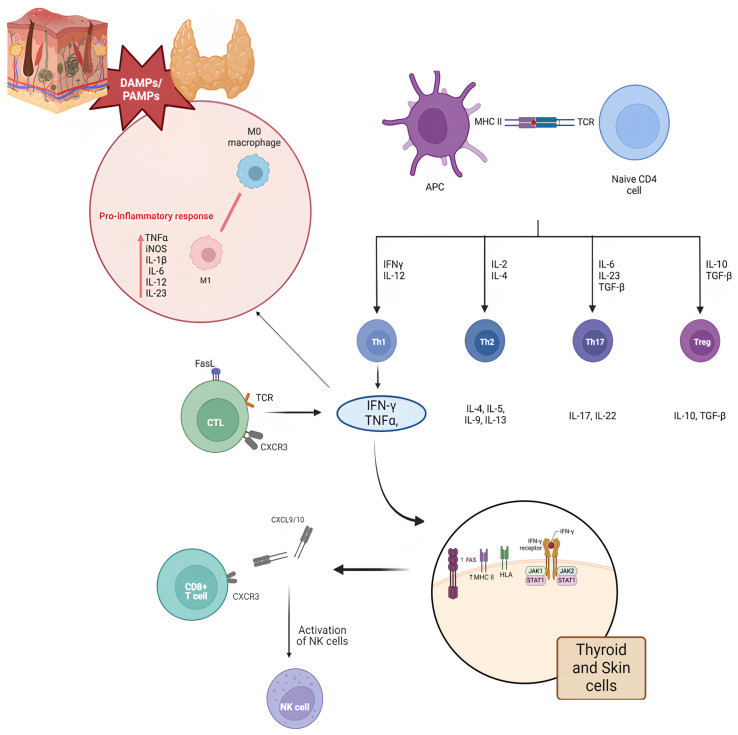
Possible triggers and cytokine pathways shared in the autoimmune response between skin diseases and thyroiditis (created with biorender.com).

Figure 1 depicts a schematic representation of the potential triggers and cytokine patterns implicated in the pathogenesis of cutaneous and thyroid autoimmune disorders.

Melanocytes, keratinocytes, and thyrocytes, when subjected to stress, release signal molecules such as HSP70 and HSP60. These molecules activate TLR (Toll-Like Receptor) receptors present on the surface of white blood cells, triggering a cascade of immune events. CTL cells (cytotoxic T lymphocytes) attack the target cells, guided by signaling molecules such as CXCL9 and CXCL10. The elimination of target cells occurs via perforins or Fas receptor activation. The release of interferon gamma (IFN-γ) by CTL cells amplifies the inflammatory response, creating a vicious circle. The immune memory is fuelled by the activation of CD8+ T cells and tissue-resident T cells, increasing the risk of relapse. T helper cells, stimulated by cytokines such as IL-1β and IFN-γ, differentiate into Th1 and Th17, in turn releasing IFN-γ, TNF-α, and IL-17. IFN-γ and TNF-α act synergistically, increasing the expression of molecules such as FAS and MHC-II on the surface of the involved cells, making them more vulnerable to attack. In addition, they induce the transformation of macrophages into M1, which produces pro-inflammatory cytokines such as IL-1β, IL-6, IL-12, IL-23, and TNF-α, further fuelling the inflammatory response. The presence of common antigens between melanocytes and thyrocytes could explain the simultaneous involvement of both tissues in autoimmune attack.

## 2. Discussion

### 2.1. Psoriasis

Psoriasis is a chronic, immune-mediated skin disorder characterized by the accelerated proliferation and aberrant differentiation of keratinocytes, resulting in the formation of distinctive, erythematous plaques with silvery scales. Affecting approximately 2–3% of the global population, Psoriasis significantly impacts patients’ quality of life and is often associated with comorbid conditions such as psoriatic arthritis, cardiovascular diseases, or metabolic syndrome. The pathogenesis of Psoriasis is multifactorial, involving a complex interplay between genetic predisposition, environmental triggers, and immune dysregulation. Key genetic loci, including *PSORS1* on chromosome 6p21, have been implicated in disease susceptibility, while environmental factors such as trauma, infections, and certain medications can exacerbate or precipitate psoriatic lesions [4].

However, from an immunological point of view, Psoriasis is predominantly driven by the activation of dendritic cells, T cells, and the subsequent production of pro-inflammatory cytokines, notably tumor necrosis factor-alpha (TNF-α), interleukin-17 (IL-17), and interleukin-23 (IL-23). These cytokines orchestrate a sustained inflammatory response within the skin, promoting keratinocyte hyperproliferation and altered epidermal differentiation [5].

Numerous studies have explored the relationship between Psoriasis, including Psoriatic Arthritis, and Hashimoto’s Thyroiditis (HT), yielding mixed results. Bianchi et al. conducted a retrospective study revealing an increased prevalence of thyroid peroxidase antibodies (TPO Ab) in patients with psoriatic arthritis compared to controls, and these patients also had a higher average thyroid volume as measured by ultrasound. Antonelli et al. [6] similarly observed a significantly higher prevalence of TPO Ab and a greater frequency of hypo-echogenic thyroids on ultrasound among psoriatic arthritis patients. They also noted that women with psoriatic arthritis had a significantly higher frequency of subclinical hypothyroidism, especially those with longer durations of Psoriasis. However, their study found no significant difference in the overall prevalence of subclinical hypothyroidism and hypothyroidism compared to controls.

A prospective study [7] that aimed to compare the incidence of thyroid diseases over six years found a significant increase in subclinical hypothyroidism, TPO, and thyroglobulin antibody (Tg Ab) positivity, and thyroid ultrasound hypo-echogenicity among psoriatic arthritis patients, particularly women.

Gul et al. [8] compared patients with Psoriasis but without arthritis to age and gender-matched controls with tinea pedis, finding no significant increase in TPO and Tg Ab prevalence in Psoriasis patients. Another prospective study [9], including 114 patients with Psoriasis (30 of whom had arthritis), compared to age and BMI-matched controls, also found no significant difference in TPO or Tg Ab positivity or in the prevalence of subclinical hypothyroidism and hypothyroidism. Furthermore, the severity of Psoriasis, assessed by the PASI score, did not influence the prevalence of thyroid autoimmunity. In contrast, a large retrospective database study including over 800,000 individuals demonstrated a significant association between Psoriasis and HT, independent of age, gender, psoriatic arthritis, and the use of systemic antipsoriatic agents.

In Alidrisi et al.’s study [10] a significantly higher prevalence of TPO Ab, Tg Ab, hypo-echogenicity, pseudo-nodularity, and increased vascularity was found in patients with Psoriasis. The prevalence in Psoriasis versus the control was found for TPO Ab (25.0% vs. 9.3%, *p* = 0.02), Tg Ab (30.4% vs. 11.1%, *p* = 0.01), hypoechogenicity (30.4% vs. 9.3%, *p* = 0.02), pseudo-nodularity (16.1% vs. 0%, *p* = 0.002), and increased vascularity (35.7% vs. 5.6%, *p* = 0.001). Obese Psoriasis patients with diagnosis onset ≥40 years were significantly more likely to have positive TPO Ab.

Current therapeutic strategies for Psoriasis encompass a range of modalities, from topical agents and phototherapy to systemic treatments and biologics targeting specific immune pathways. Despite advancements in understanding the molecular mechanisms underpinning Psoriasis and the development of targeted therapies, challenges remain in achieving long-term disease control and addressing the heterogeneity in treatment response among patients.

Ongoing research aims to further elucidate the genetic and immunological underpinnings of Psoriasis, identify novel therapeutic targets, and develop personalized treatment approaches to optimize patient outcomes.

### 2.2. Lichen Planus

Lichen Planus has been associated with a number of autoimmune disorders like miastenia gravis, Psoriasis, Sjogren’s syndrome, coeliac disease, and ulcerative colitis.

Oral Lichen Planus (OLP) is a chronic autoimmune disease with a prevalence of 1–2% in the general population, and which most commonly affects middle-aged and elderly females. The specific etiology and pathogenesis of OLP remain unclear. Various factors such as immune dysregulation, genetic predisposition, psychological factors, and infectious precipitants play crucial roles in the development of OLP. Amato-Cuartas et al. found that the prevalence of hypothyroidism in Colombian patients with OLP was almost ten times the value in the entire study population (35.7% vs. 3.95%). [11] The same result but with slightly lower values was found by Lo et al. [2] (14.3 % vs. 2%). Lo Muzio et al. [12] demonstrated that the prevalence of HT in the OLP patients was 14.3% in Italy, whereas the prevalence in the general population was only 1%. These data were found to be statistically significant (*p* < 0.0003, OR = 14.29, 95% CI = 1.9, 106.2). Tang et al. reported that the prevalence was 12.14% in East China [13]. Li et al. [14] conducted a meta-analysis with the articles published between 2010 and 2016, hypothesizing common etiopathogenetic mechanisms. According to Zhou et al. [15], in a Chinese case–control study, the prevalence of thyroid disease in these patients was significantly higher than the control group, showing an increased risk of HT and thyroid nodule. Zhang et al. [16] showed that the prevalence of HT in all patients with OLP was 39.68%, of which the prevalence in females was 46.24% while in males it was 19.67% (*p* < 0.01). The titers of the HT autoantibodies in females were higher than those in males (*p* < 0.01). However, HT prevalence and autoantibody levels in patients with OLP were not relate to the clinical manifestations. Alikhani et al. reported that the blood levels of TPOAb were significantly correlated with an increased risk of erosive OLP [17].

From a histological point of view it is possible to observe a connection between these two pathologies; while HT is characterized by lymphocytic infiltration and fibrosis of the thyroid gland, in OLP there is necrosis of the basal cell layer and subepithelial band-like infiltration of lymphocytes, mostly T lymphocytes. Therefore, both OLP and HT involve inflammatory infiltration, confirming the hypothesis of an autoimmune mechanism underlying the two pathologies.

Considering the already known correlation between autoimmune thyroid disease (AITD) and autoimmune skin disease, it has been hypothesized that AITD may result in the production of antigens within the damaged thyroid tissue, leading to the activation of antigen-specific B cells that subsequently release antibodies. Alikhani et al. correlated the severity of OLP lesions to TPOAb levels, which are the best serological markers for diagnosing HT, while TGAbs have less sensitivity and specificity. Although keratinocytes do not express TPO, circulating TPOAB may cross-react with unknown surface proteins and trigger Cd95-(Fas/Apo-1) mediated apoptosis [18]. The internalization of apoptotic bodies subsequently leads to the activation of T cells. Most T cells adjacent to damaged basal keratinocytes are CD8+ cytotoxic T cells, which are the same cells that release perforin and granzyme, damaging the thyroid cells of the patients affected by HT. Lo Muzio et al. [12] also suggested the possibility that thyroid antibodies could have the ability to unmask epitopes in keratinocytes.

Previous studies have shown an increased IFNγ/IL-4 ratio, indicating that OLP is TH1-based; the same ratio was found in HT patients. Therefore, it was demonstrated that both OLP and HT are predominantly Th1-type cytokine diseases. Furthermore, the TPOAb level was associated with an increased production of Th1 cytokines [18].

Other types of cells that are found to be increased in both diseases are Th17 cells, whose interleukins promote T cell proliferation and thyroid autoantibody production, leading to thyroid tissue damage, and Th22 cells.

Environmental factors play an important role in the pathogenesis of OLP and HT. For example, the relationship between smoking and OLP/HT is controversial and unclear. Recent studies have suggested its protective effect for OLP and thyroid diseases. Carlè et al. showed that smoking cessation is followed by a sharp but transient rise in the incidence of HT [19].

It has been supposed that OLP and HT share some common immunological triggers and genetic susceptibility. Human leukocyte antigen (HLA) is the main histocompatibility complex in humans and its polymorphism is involved in many diseases. Data on HLA haplotypes in HT showed an association of atrophic HT with HLA-DR3 and goitrous HT with HLA-DR5. Hawkins et al. found that HLA-DRw9 is associated with Graves’ disease and HT in Chinese populations [20]. This prevalence was confirmed by Lin and Sun’s work [21]. Other studies have shown that HT is associated with HLA-DR3 alleles in Caucasians [22], and an association with HLA-DQw7 has been reported too. The same study also demonstrated that the gene for cytotoxic T lymphocyte antigen 4 (*CTLA-4*) is able to confer susceptibility to HT. The presence of *CTLA-4* downregulates T cell activation, and therefore polymorphism or mutations altering *CTLA-4* expression or function could result in exaggerated T cell activation. The main infectious agents found in both pathologies are hepatitis C virus [23], human herpesvirus 6 [24,25], and *Helicobacter Pylori* [26], and less evidence was highlighted with Epstein–Barr virus and Cytomegalovirus. This plays an important role in considering the structural comparability between microbial antigens and human autoantigens. So, in genetically predisposed subjects, a defensive immune response may be directed against autoantigens. According to this study, hypothyroidism did not confirm its prevalence, even if previous studies have indicated that HT was the most common cause of hypothyroidism.

Vitamin D has a beneficial effect on the immune system, so insufficient intake is involved in many autoimmune diseases. Recent studies suggest that vitamin D deficiency is associated with thyroid autoimmunity, and therefore a lack of this vitamin in patients with OLP and HT may promote Th1 lymphocyte activity and its cytokine expression. It is well known that stress can induce a variety of immunological changes that are associated with several autoimmune diseases. Acting on neuromodulation and endocrine regulation can affect the Th1/Th2 balance [27]. Not only stress but also anxiety and depression [28] are correlated with OLP, highlighting the importance of the mental state.

Looking at the epidemiological data on the prevalence of these pathologies in female subjects, the possible correlation with sex steroid hormones was studied. It has been shown that the prevalence of OLP in perimenopausal women [29] is related to hormonal fluctuations, while it has been confirmed that estrogen is closely involved in autoimmune thyroid disease. Furthermore, the estrogen receptor expression is increased in HT patients [30]. These results suggest not a direct relationship between estrogen and OLP, but its involvement in the pathogenesis of OLP and HT and their co-occurrence.

Wu et al. [31] hypothesized that OLP might be secondary to HT in some cases of OLP and HT co-occurrence. Therefore, a routine screen for thyroid diseases is recommended in OLP patient, especially in women over 40 years of age. Lo Muzio et al. confirmed that hypothesis, arguing that in HT patients, circulating thyroid antibodies could also contribute to the triggering of an autoimmune response in the oral mucosa or skin, leading to the development of OLP lesions. Furthermore, patients suffering from HT should be informed about the risk of developing OLP lesions.

### 2.3. Atopic Dermatitis

Atopic Dermatitis (AD) is a chronic, relapsing, inflammatory skin disease that affects up to 5% of the adult population and up to 10% of children. It is characterized by itchy papules-papulovesicular, erythema, excoriations, and lichenified plaques. Interactions between genetic mutations, skin barrier dysfunction, immunologic aberrations, and environmental factors are the major pathogeneses of AD [32]. The mutation of FLG, encoding the protein filaggrin, is frequently found in patients with AD, leading to skin barrier dysfunction. Other genes involved are *SPINK 5* (serine peptidase inhibitor Kazal-type 5), encoding for a specific antiprotease, and those related to the regulation of immune response (IL-4 and its receptor, IL-13, and RANTES chemokine).

According to the counter-regulation of T-helper 1 and 2, it is expected that Th1-type autoimmune diseases, like thyroid autoimmunity, and Th2-mediated allergic disease should be presented in different types of populations. Although thyroid autoimmunity has been regularly associated with chronic urticaria in children, the correlation of thyroid autoimmunity and Atopic Dermatitis has not yet been fully investigated. Additional lymphocyte subsets like Th17 cells and regulatory T-cells (T reg) have been indicated as a common link between atopy and autoimmunity [33]. Indeed, T-reg cells are essential for the maintenance of immune homeostasis and the prevention of autoimmunity [34]. Furthermore, a deficient number of functions of these cells has been demonstrated in atopy patients [35].

There is increasing evidence of an association with several non-atopic conditions such as Crohn’s disease, ulcerative colitis, coeliac disease, Alopecia Areata, and Vitiligo, but also with cardiovascular diseases [36] and neuropsychiatric disorders [37]. Inflammatory bowel disease (IBD) occurs due to abnormal regulation of Th1 and Th2 cells by T reg cells, similar to AD [38].

The association between AD and thyroid disease was investigated by two important studies. The first one, conducted by Wu et al., found a higher prevalence of thyroid disease in AD patients than in control subjects (OR 1.29; 95% CI, 0.99–1.46), but the data did not achieve statistical significance [39].

Pedullà et al. [40] showed a significantly increased prevalence of thyroid autoimmunity in children affected by Atopic Dermatitis (9.52%) compared with healthy controls. Furthermore, the frequency of thyroid autoimmunity was higher among children with IgE-mediated AD (18.51%) than non-IgE-mediated AD (4.3%), suggesting that atopy and thyroid autoimmunity could be two outcomes of dysregulated immunity. Therefore, they found a significant association between the frequency of atopy and thyroid autoimmunity (RR 4.1 95% CI 1.42–13.06). Another study was conducted by Pedullà et al., which also found a significant prevalence of thyroid autoimmunity in atopics (13.67%) as compared with non-atopics (2.7%) in children with AD and a significant association between thyroid autoimmunity and atopy (OR = 5.76, 95% CI: 1.71–19.35) in all children with skin disease (AD, urticaria, and Alopecia Areata) [41]. The same prevalence but with lower results has been found in several studies (Marwaha et al. (1.6% in 6183 girls) [42] and Jaksic et al. (0.35% in 5462 children) [43]). An Indian cross-sectional study, including 62 children with AD, showed a prevalence of 18,9% based on the presence of anti-TPO antibodies, but only half of these had impaired thyroid function [44]. A limitation of the latter study was the non-availability of the thyroglobulin antibody and TSH receptor antibody. The different results in previous studies are likely due to the heterogeneity with respect to characteristics of the study population, like age and race.

A reverse analysis was performed by Zhang et al. [45] that analyzed 217 Chinese patients with positive Tyroglobulin antibody (TgAb) and/or TPOAb, funding the prevalence of allergic rhinitis, CSU, and AD. As regards Atopic Dermatitis, they present a double value compared to that of one of the controls. Risk factor analysis also identified thyroid autoantibodies TgAb or TPOAb as potential risk factors for the diseases mentioned above (allergic rhinitis, AD, or CSU). Some hypotheses see the formation of immune complexes in AITD patients by autoantibodies and thyroid antigens (for example, TPO). These complexes, binding the Fc receptor on mast cells and basophils, induce the activation of allergy responses in the skin [46]. The inflammatory process in AD is biphasic. In the acute phase, Th2 cytokines, like interleukin-4, interleukin-5, and interleukin-13, are more involved, while in the chronic one, there is an increase in g-interferon, interleukin-5, interleukin-12, and GM-CSF, characteristic of Th1 and Th0 immune responses. The inflammatory response that occurs in AD is mediated by the Th2 lymphocyte and its cytokines, which can lead to an IgE response to different environmental antigens. This could explain why AD patients are highly sensitized to airborne allergens and frequently present allergic rhinitis and asthma, too [47]. Interestingly, a high percentage of AD patients present IgE autoreactivity to a vast spectrum of human proteins, expressed in a variety of cell and tissue types [48].

Autoimmune diseases present a multifactorial etiology, but one of the principal causes is that an infection or exposure to a cross-reactive antigen leads to an immune dysregulation autoimmunity [40]. Furthermore, IgE auto-antibodies have been detected against more than 140 self-binding antigens in AD and against TPO in chronic spontaneous urticaria [49].

The final message is to evaluate TPO and TG autoantibodies in atopic children and adults affected by skin disease, as they could have concomitant TA.

Oxidative stress has also been implicated in the pathogenesis of AD. Especially during AD exacerbation, there is an oxidative imbalance with a decreased action of antioxidant systems. Chronic skin inflammation is associated with the overproduction of reactive oxygen species (ROS), but exogenous factors (solar radiation, pollution, psychological processes) also make their contribution. Many urinary and serum biomarkers have been evaluated, such as malondialdehyde (MDA), urinary 8-hydroxydeoxyguanosine (8-HdG), glutathione (GSH), 4-hydroxy-2-nonenal (HNE), and nitric oxide (NO), but these data are still incomplete, because of several limitations of the available literature, the small size of the study populations, and the limited number of included countries [50].

### 2.4. Alopecia Areata

Alopecia Areata is a common chronic tissue-specific autoimmune disorder affecting up to 2% of the general population, characterized by patchy, recurrent, and non-scarring loss of hair in the anagen phase [51]. The clinical manifestations vary from small well-defined patches to more diffuse or total hair loss of the scalp, alopecia totalis, or hair loss of the entire body, alopecia universalis. In a number of patients it can be persistent, and more frequently when there is an extended involvement [52]. It is easy to understand how alopecia negatively impacts the quality of life of these patients, leading to psychological disorders like anxiety and depression [53]. AA is an inflammatory T-cell-mediated autoimmune reaction against an unknown autoantigen of anagen-phase hair follicles. It has an unpredictable course and no significant predilection to sex, age, and race. The etiology is not fully understood, but causative factors could be found among genetic susceptibility, autoimmunity, and environmental factors, like infection and vaccines [54]. The genetic basis is explained by a higher familial occurrence, with a positive family history in 10–42% of patients [55], while the estimated lifetime risk in the general population was 2% [56]. No monogenic cause of AA has been detected. Recently, a genome-wide analysis of copy number variants (CNVs) identified duplications in melanin-concentrating hormone receptor 2 (*MCHR2*), suggesting an important role of the melanin-concentrating hormone (MCH) in the development of this cutaneous disease. Petukhova et al. [57] found two genes (*ATG4B* and *SMARCA2*) involved in autophagy and chromatin remodeling. Genetic studies have also identified the potential involvement of syntaxin-17 (*STX17*) and peroxiredoxin-5 (*PRDX5*) genes. Therefore, the dysregulation of *PRDX5* causes cells affected by oxidative stress to survive and express damaged self-antigens [58].

AA is associated with a vast group of autoimmune disorders: diabetes mellitus, lupus, celiac disease, and HT. The most established associations are with hypothyroidism and Vitiligo [59].

Non-scarring alopecia is part of the diagnostic criteria for systemic lupus erythematosus in 2019 by the American College of Rheumatology and the European League against Rheumatism [60]. According to the autoimmunity theory of AA, the infiltration of lymphocytes with CD4+, CD8+ T cells, and Natural killer cells was found in a biopsy specimen of affected hair follicles of AA patients. Various studies have demonstrated a higher frequency of AA in thyroid disease patients. Milgraum et al. [61] found an apparent association between AA and thyroid disease. Later, Lewinski et al. [62] confirmed this prevalence. In contrast with these studies, Puavillai et al. [59] demonstrated that the prevalence of thyroid disease is relatively low, only 7.2%, with a non-statistically significant difference compared to controls. Seyrafi et al. [63] found thyroid disease in 8.9% AA patients, while Bakry et al. [64] showed that 16% of AA Egyptian patients had hypothyroidism. Park et al. [65], who analyzed 1408 patients, and Lee et al. reported an increased incidence of thyroid autoimmunity and dysfunction in severe AA patients, compared to the average population [66]. Contrasting results were obtained between levels of anti-TPO and TSH and predilection of male to female [47,67,68]. However, Gonul et al. [69] highlighted a correlation between thyroid antibody levels and increased disease duration. Different geographical locations reported a different incidence value of thyroid dysfunction, as follows: Singapore, 2.3%; Beijing, 6.7%; Spain, 22%; India, 18.3%; North America, 14.6% [70].

The most accepted hypothesis, underlying the pathology, is the collapse of the immune privilege of the hair follicle, which is associated with the secretion of INF-g and the upregulation of NKG2D ligands, MHC I and MHC II molecules, and chemokines like IL-2, IL-15, and CXCLs. On the other hand, there is a reduction in local immunosuppressant molecules (TGF-b1, IL-10, a-MSH, and VIP) [71]. Recent studies have pointed out the fundamental role of IFN-g and CD8+ NKG2D+ T cells in AA pathogenesis [72]. Indeed, the intravenous injection of IFN-g could be associated with AA-like hair loss in young C3H/HeJ mice, while IFN-g-deficient mice were found to be resistant [73]. Similar studies showed that CD8+ NKG2D+ T cells produced IFN-γ with JAK1 and JAK2 pathways, while IL-15, binding to the surface of these cells, increased the release even more [58].

The role of oxidative stress cannot be overlooked. It induces an upregulation of NKG2D, leading to the collapse of the hair follicle immune privileged site. In blood cells and lesions of AA patients have been evaluated, a reduction in erythrocyte superoxide dismutase (SOD) and glutathione peroxidase (GSH-Px) activities, as well as an increase in malondialdehyde (MDA) levels [74,75] has been noted. A recent meta-analysis [76] demonstrated that the oxidative damage products (MDA and NO) and the total oxidant capacity (TOC) were significantly raised in AA patients. This occurred more frequently in the severe form of AA compared to the mild/moderate form, suggesting a proportional relationship. Oxidative stress promotes MICA, MHC class I chain-related A, and the expression of the hair follicle cells [77]. Normally, these sites are usually MICA-negative, while AA shows positive MICA expression, leading to the activation of the innate immune system.

Lousada et al. [78] and Pinto et al. [79] showed the role of microbial dysbiosis in hair loss, revealing a significant increase in *Propinebacterium acens* and a decrease in *Staphylococcus epidermidis* in AA patients. On the other hand, the analysis of the intestinal microbiota indicated various components of fecal microbiota, mainly *Parabacteroides distasonis* and *Clostridiales vadin BB60* [80].

In conclusion, it is necessary to underline the importance of evaluating thyroid function and antibodies irrespective of clinical status in AA patients.

### 2.5. Vitiligo

Vitiligo is a chronic autoimmune disorder characterized by multifocal, well-defined, achromic patches on the skin. It occurs with a frequency of 0.1–2.0% in various populations [81]. Underlying this clinical picture, there is depigmentation of the skin due to the destruction of melanin produced by melanocytes, the pigment-producing cells within the epidermis. Since the case series reported by Steve [82], several publications have suggested a potential association between Vitiligo and other autoimmune diseases, particularly autoimmune thyroid disorders (AITD). This correlation has been validated in several metanalyses [83,84].

Although genetic predisposition and autoimmune etiology are likely to contribute to the observed co-occurrence of these diseases, the precise immunological process behind this comorbidity is still not fully understood. Possible common pathogenetic mechanisms will be discussed in the following sections, such as genetic susceptibility, immunologic abnormalities, and oxidative stress.

Studies have identified a link between genes associated with AITD and Vitiligo susceptibility. This genetic association has also been demonstrated in close relatives, regardless of whether or not those relatives have Vitiligo themselves, suggesting a potential hereditary predisposition, where shared susceptibility genes likely play a contributory role [85,86]. Early investigations focused on HLA loci, the major histocompatibility complex (MHC) on chromosome 6. Genome-wide association studies (GWAS) have since identified specific loci linked to AITD, with HLA-DR3 showing the strongest connection. Conversely, generalized Vitiligo is associated with HLA-DR4, as reported by Foley and colleagues [87], while Liu et al. [88] performed a meta-analysis of eleven studies on HLA class I serotypes and discovered a strong association between Vitiligo and HLA-A2. More recent studies suggest that HLA DQ* polymorphism may confer susceptibility to AITD and early-onset Vitiligo. [89,90,91].

Over the years, researchers recognized the potential involvement of non-MHC gene associations, which have received widespread independent confirmation, including by unbiased GWAS. While several genes have been linked to AITD, only a limited number have been confirmed in multiple independent studies. These include genes involved in both immune regulation, such as *CTLA-4* and *PTPN22*, and thyroid function, like thyrosinase (TYR), thyroglobulin (Tg), and Thyroid Stimulating Hormone Receptor (TSHR).

*CTLA-4* is a protein-coding gene; it is an inhibitory receptor belonging to the CD28 immunoglobulin subfamily, expressed primarily by T-cells. *CTLA-4* plays a key role in regulatory T cells and central tolerance, because of its importance in the maintenance of peripheral tolerance, [92].

Direct association between Vitiligo and *CTLA-4* appears to be weak, as demonstrated by a meta-analysis by Birlea et al. [93]. Indeed it may be secondary, driven by primary genetic association with other autoimmune diseases that are epidemiologically associated with Vitiligo, such as AITD.

Similarly to AITD, Vitiligo exhibits a non-MHC gene association with *PTPN22*, the gene encoding lymphoid protein tyrosine phosphatase (LYP). *PTPN22* has well-established genetic links to various autoimmune disorders, including AITD. Notably, a meta-analysis by Wu et al. [94] demonstrated that this association is particularly strong in European Caucasians. Further supporting this ethnicity-specific effect, another meta-analysis revealed a significant association between the PTPN22 +1858C→T polymorphism and Vitiligo in European populations (odds ratio = 1.53) but not in Asian populations (odds ratio = 0.59) [95]. These findings collectively suggest that the *PTPN22* specifically increases susceptibility to Vitiligo and AITD in individuals of European white descent.

Furthermore, among these, there are thyroid-specific genes such as those coding for TYR, Tg, and TSHR [96], suggesting that these loci may be relevant principally to patients with isolated AITD, and that broader autoimmunity susceptibility genes may be of greater importance in patients with concomitant generalized Vitiligo and AITD. In addition, an autoimmunity susceptibility locus (AIS1) was identified by genome-wide linkage analysis on chromosome 1 in families characterized by Vitiligo and HT [97]. Lu et al. [98] performed a bioinformatics analysis to search for possible biomarkers of the Vitiligo–HT comorbidity. Their study focused on immune-related genes, performing differential expression analysis between Vitiligo and HT datasets. Four key hub genes were revealed among the IRDEGs (*IFNG, IL1B, STAT1*, and *CXCL10*):

- *IFNG*: encodes IFN-γ, an inflammatory cytokine that activates immune cells.
- *STAT1*: encodes a signaling protein involved in the IFN-γ response.
- *IL1B*: encodes interleukin-1 beta, a pro-inflammatory cytokine that recruits immune cells.
- *CXCL10*: encodes the chemokine CXCL10, which attracts T cells.

These genes and their interactions might contribute by influencing both innate and adaptive immune responses. The observed simultaneous damage to the skin and thyroid could be linked to alterations in T-cell subtypes, macrophage polarization, and the presence of shared target antigens. Among the identified genes, *CXCL10* emerged as the most promising candidate for diagnosis based on ROC curve analysis. In practice, damage-associated molecular patterns (DAMPs) and pathogen-associated molecular patterns (PAMPs) activate innate immune cells, which in turn promote the adaptive T-cell response. Infiltrating CD8+ T cells are responsible for cytotoxic destruction in both Vitiligo and AITD [99,100,101]. These T cells secrete type 1 cytokines, particularly IFN-γ and tumor necrosis factor-alpha (TNF-α). The recruitment of CD8+ T cells to the skin is facilitated by the CXCR3 receptor binding to CXCL9 and CXCL10 chemokines. Importantly, the IFN-γ activates the JAK/STAT pathway in keratinocytes, which induces the production of CXCL9 and CXCL10, creating a positive feedback loop that perpetuates the inflammatory response.

The current literature displays evidence suggesting a strict interplay between oxidative stress and the immune system in both Vitiligo [102,103]) and AITD [104]. An imbalance between oxidants and antioxidants is observed at different stages and in different types of autoimmune diseases. Chronic inflammation can lead to the accumulation of local and systemic reactive oxygen species (ROS).

The thyroid gland uses ROS in its hormone production and releases enzymes that promote ROS generation; therefore, a critical role is played by the immune defense system and non-enzymatic antioxidants that counteract as a buffer to ensure homeostasis. Due to its dependence on ROS for function, the thyroid is especially vulnerable to oxidative stress and an excess of free radicals can jeopardize thyroid functionality [105].

On the other hand, Melanin synthesis by melanocytes represents an intracellular stressor, producing ROS. Antioxidant defenses, such as the Nrf2/ARE pathway and the Hsp70 chaperone system, represent the main defense for stressed melanocytes from cell death. The presence of autoantigens, shed by stressed melanocytes, triggers the innate immune response, bridging the gap between oxidative stress and the adaptive immune system. In conditions like Vitiligo, for instance, Heat Shock Protein 70 (Hsp70) facilitates the activation and maturation of Dendritic Cells (DCs), which then present autoantigens to CD8+ T cells. These activated CD8+ T cells, supported by Th17 cells, orchestrate the destruction of melanocytes [102].

A recent study by Li et al. [106] proposes a novel link, suggesting that the shared origin of melanin and thyroid hormones from tyrosine might contribute to this comorbidity. Thus, melanocytic and thyroid systems might interact, since the same oxidative stress that damages the thyroid might also trigger the immune system to attack pigment cells in the skin, causing Vitiligo. At the same time, in patients with thyroid autoimmunity, increased ROS levels have been demonstrated which might contribute to modifying tyrosinase or other melanogenic proteins into neoantigens, leading to the appearance of Vitiligo.

### 2.6. The Role of Immune Checkpoint Inhibitors (ICIs)

ICIs have significantly advanced cancer treatment by enhancing the immune system’s ability to recognize and attack tumor cells. However, the underlying mechanism of ICIs can potentially induce autoimmune reactions, due to the disruption of immune tolerance, leading to the activation of self-reactive CD8+ T cells [107]. When ICIs block the PD-1/PD-L1, CTLA-4 pathways, and so on, T cells are activated, resulting in increased cytokine production and signaling through the JAK-STAT pathway in targeted end-organ cells. The loop involving B cells and plasma cells in the production of pro-inflammatory cytokines (IL-6 and TNF-α) and auto-antibodies, which can form immune complexes or bind directly to tissues, contributes to the development of autoimmune diseases, including thyroiditis and skin conditions. Furthermore, ICIs can also affect regulatory T and B cells (Tregs and Bregs), which typically maintain tolerance, reducing their functions or numbers. Dougan et al. [108] highlighted a correlation between individuals with certain human leukocyte antigen (HLA) alleles and a high risk of developing ICI-induced autoimmune diseases.

Thyroiditis is a common autoimmune complication associated with ICI therapy. It can present as hypothyroidism or hyperthyroidism, and its diagnosis often requires thyroid function tests. Skin manifestations of ICI-induced autoimmune reactions are also frequent and can range from lichen planus to severe bullous diseases. Laboratory tests and histopathological examination are essential for confirming these diagnoses.

While the risks of autoimmune reactions associated with ICIs are significant, their benefits in cancer treatment often outweigh these drawbacks. Careful monitoring and the early detection of autoimmune complications are crucial for managing these adverse effects. In some cases, immunosuppressive therapies, such as intravenous immunoglobulin (IVIG), may be necessary to treat severe autoimmune reactions.

## 3. Conclusions

This review highlights the potential for shared underlying mechanisms in various autoimmune diseases. The precise way in which anti-thyroid antibodies contribute to the development of autoimmune skin diseases and vice versa is not fully understood, but the observed co-occurrence highlights the complex interplay between genetics, environment, and the immune system and points towards an autoimmune shared trigger.

Some researchers have proposed potential explanations for this connection. The shared origin of the skin and the thyroid from the ectoderm suggests potential similarities in their immunological profiles and pathways. Both organs express major histocompatibility complex (MHC) molecules, which are crucial for the immune system’s ability to differentiate self from non-self. The presence of MHC molecules allows immune cells to recognize and tolerate healthy tissues while identifying and eliminating foreign invaders. Additionally, both skin and thyroid tissues express various cytokines and chemokines, signaling molecules that orchestrate immune responses. These shared features hint at a vulnerability in the context of autoimmunity. If a breakdown in immune tolerance occurs, the immune system, primed to recognize certain antigens in the context of the shared origin, could launch an attack on both the skin and the thyroid.

Given the complex interplay between these conditions, a multidisciplinary approach is essential for optimal patient care. A number of studies have indicated the potential value of preventive thyroid antibody and functionality testing in patients with autoimmune skin diseases. It may reasonably be proposed that this practice should be incorporated into the routine diagnostic work-up of such diseases. Clinicians may include the measurement of anti-TPO and anti-thyroglobulin (anti-TG) antibodies, along with TSH, FT3, FT4 levels, and a thyroid ultrasound exam, as part of routine monitoring for thyroid function in patients with autoimmune skin diseases, given the frequent association with thyroid disorders. Future research should focus on identifying targeted therapies and personalized treatment strategies for patients with these coexisting conditions, acting on IL-23/Th17, CXCL9 and CXCL10, JAK/STAT, and CTLA-4. Immunologists, Dermatologists, and Endocrinologists should inquire about a family history of autoimmune diseases and screen patients for potential co-occurring conditions. Future research directions could focus on elucidating the specific immunological pathways involved, identifying novel biomarkers for early diagnosis and potential therapeutic targets, and developing personalized treatment strategies.
